# Negotiating Female Genital Cutting in a Transnational Context

**DOI:** 10.1177/1049732320979183

**Published:** 2021-01-10

**Authors:** R. Elise B. Johansen, Salma A. E. Ahmed

**Affiliations:** 1 Norwegian Centre for Violence and Traumatic Stress Studies, Oslo, Norway; 2 Federal Ministry of Health, Khartoum, Sudan

**Keywords:** female genital cutting, migration, decision-making, Sudan, Somalia, Norway, qualitative

## Abstract

In this article, we explore migrant Somali and Sudanese women’s reflections and decision-making regarding female genital cutting in a transnational context wherein women are compelled to maneuver between contradictory social norms. These include traditional norms, which consider the practice to be associated with socially acceptable sexuality and reproduction, and international norms, which consider the practice to be a violation of sexual and reproductive rights. Our analysis builds on data from in-depth interviews with 23 women of Somali and Sudanese origin residing in Norway. Informed by three central theories of change, we categorize women along a continuum of readiness to change ranging from rebellious women eagerly pursuing the abandonment of female genital cutting and adopting international norms regarding the practice, to women supporting the practice and its traditional meanings. Ambivalent contemplators were placed in the middle of the continuum. Women’s positioning was further interlinked with social networks and perceived decision-making power.

## Introduction

In this article, we explore social and cultural factors that drive or obstruct readiness to change female genital cutting (FGC) among migrant Somali and Sudanese women in Norway. In this transnational context, women maneuver between two contradictory and incompatible clusters of meaning: traditional norms and international norms. Traditional norms refer to the Somali and Sudanese understanding of FGC as a prerequisite for women’s sexual morality, marriageability, and proper fertility (Abdalla, 1982; Abusharaf, 2005). In contrast, international norms, as formulated by the United Nations, define FGC as a violation of human rights, particularly women’s sexual and reproductive rights (World Health Organization, 2008). As traditional norms related to FGC are dominant in some women’s countries of origin and international norms are dominant in Norway, we also explore the social factors of women’s meaning-making in terms of ethnicity and place of residence. Finally, we explore perceptions of decision-making power, as migration implies that those traditionally involved in FGC decision-making—that is, generally elder female family members—are spread between different countries and thus adhere to different clusters of meaning that are influenced by different legal and sociocultural factors.

We open this exploration with the description of a narrative extracted from the interview with one of our study participants that illustrates some of the complexities of FGC meaning and decision-making when migration is a factor. The woman in question was born in Somalia but was subjected to FGC in a bordering country at the age of 7 years while spending a few months there during her migration to Norway. Her father was already settled in Norway and had been granted the right to bring along his wife and daughter through a system of family reunification. To get the papers sorted for their resettlement, the girl had thus moved with her mother and siblings to the country in question because it hosted a Norwegian embassy. There, they moved in with relatives, including the mothers’ sister. A discussion about whether or not to subject the girl to FGC before continuing to Norway ensued. The father argued against FGC, considering it unnecessary now that the girl was going to live in a country without the social pressure for FGC that was present in Somalia. Although the girl’s mother agreed with the father, the girl’s aunt did not. She found it important to have the girl circumcised prior to migration as she knew the practice was illegal in Norway. She also found that the migration necessitated FGC as a measure to safeguard the girl’s virginity and morality in a country she associated with sexual freedom and less social control. Therefore, the aunt seduced the girl to come with her to a clinic to have it done on a day that the girl’s mother was away. When told about her sister’s act upon her return, the girl’s mother rushed to the clinic where the procedure was well underway. There, she managed to convince the provider to make a less tight closure when stitching together the labia than what is common in the infibulation type of FGC.

Growing up in Norway and inspired by her studies in health, the participant came to understand the health risks associated with FGC. Nevertheless, she was reluctant to speak critically about it with fellow Somalis for fear of negative reactions. She also supported many of the cultural values underlying the practice, including the importance of premarital virginity. In addition, she conformed to the social expectations of endogamous marriage. Upon marriage, she did not require the reopening of the closure, which is usually necessary for sexual intercourse, because of the limited closure of her FGC. However, she considered premarital defibulation to be socially impossible even in Norway, as it would be interpreted as a sign of premarital sexual engagement and thus moral laxity.

These extracts describe some of the many decisions that directly or indirectly relate to FGC and how it is negotiated in a context where one may relate to both traditional and international norms and values. Furthermore, these negotiations related to contextual factors both in one’s country of origin, country of current residence, and destination country. The way in which the girl’s aunt subjected her to FGC despite her parents’ resistance illustrates differences in decision-making power and how the constitution and relative power within a decision-making group may change in the context of migration.

Inspired by this story and others, we decided to examine FGC-related decision-making broadly rather than limiting the exploration to decisions on whether to subject girls raised in Norway to FGC. We broadened the scope to include decisions relating to physical, cultural, and social aspects of FGC. Physical aspects include decisions on whether to cut, the type of cut, and the provider as well as decisions regarding defibulation and reinfibulation (resuturing the infibulation) later in life. Decisions about the cultural aspects of FGC are related to meaning-making and the extent to which one perceives FGC in terms of traditional or international norms. As migrant women are exposed to both norm sets, the way they relate to these norms can be regarded as a choice. Framing FGC based on traditional norms would imply a perception of their own circumcision as a procedure that enhances their bodies aesthetically, morally, and sexually. In contrast, understanding FGC within an international normative framework could lead to a perception of FGC as a procedure that impairs, harms, and destroys their bodies. Meaning-making is thus deeply personal (Johansen, 2002; Lien & Schultz, 2013). Furthermore, the framing of FGC is profoundly social, as the two norm sets are dominant in different social networks. If interpreted within the traditional norms dominant in country of origin, FGC can contribute to social acceptance, including marriageability. In contrast, women with FGC may feel stigmatized and excluded vis-à-vis the Norwegian host community, as it adheres to international norms condemning FGC (Talle, 2008).

The transnational setting of the diaspora also leads to changes in the decision-making processes and distribution of decision-making power. In the country of origin, FGC decisions are commonly made by larger groups of people, in which girls’ mothers may have had limited decision-making power (Bedri et al., 2019). Furthermore, decisions have commonly been strongly influenced by community pressure (Hamed et al., 2017; Newell-Jones, 2016). In the diaspora, however, the situation differs dramatically. Family members who traditionally play a central role in decision-making often live in different countries and continents, thus altering the decision-making group and its power distribution. Furthermore, decision makers face a variety of social norms, which have been found to reduce the social sanctions of deviance (Grose et al., 2019; Wahlberg et al., 2019). In addition, migration seems to lead to increasing privatization and silencing of the topic, partly due to changing perceptions in the community and stigmatization from the host community (Johansen, 2019). This reduction of shared discourse may reduce social pressure but may also reduce the clarity of social norms, thus making decision-making more challenging.

### Understanding FGC Decision-Making and Change

To understand the interplay between personal, cultural, and social factors relevant for women’s FGC-related decision-making, we find it useful to build on insights derived from three theoretical perspectives used to understand cultural change: Gerry Mackie’s theory of FGC as a social convention (Engelsma et al., 2020; Mackie & LeJeune, 2009), Everett Rogers’ theory of diffusion of innovation (Rogers, 2010), and Bettina Shell-Duncan’s modeling of readiness to change (Shell-Duncan et al., 2011; Wander & Shell-Duncan, 2020). These approaches complement each other, with the first focusing on the power of social norms, the second on individual readiness to change, and the last adding perspectives on decision-making power and contextual factors. We briefly outline each of these frameworks below.

Mackie’s theory of FGC as a social convention has been the most popular approach over the past decades (Brown et al., 2013; Cloward, 2015; Shell-Duncan et al., 2011; United Nations Children’s Fund [UNICEF], 2005; Wahlberg et al., 2019). The main argument holds that FGC constitutes a social convention compelling people to comply irrespective of their personal attitude. As this implies an interdependency of decision-making within a group, change can only happen jointly, when a critical mass decides to abandon the practice. The critical mass then forms a tipping point persuading the rest to follow. As the importance of FGC as a social convention is closely interlinked with marriageability, abandonment requires that FGC no longer be perceived as a prerequisite for marriage.

To understand how one reaches a tipping point, however, we need a theoretical understanding of how change is initiated and builds up to a critical mass. Here, we found Rogers’ (2010) theory of diffusion of innovation useful. The model indicates that there are systematic differences in peoples’ readiness to change, categorizing five different groups: (a) Innovators are venturesome, interested in new ideas and willing to take risks, (b) Early adopters embrace opportunities for change initiated by innovators, (c) Early majority adopt new ideas before the average person, given that they see evidence of benefits of the change in question, (d) The late majority are skeptical of change and will only adopt an innovation after it has been tried by the majority, and (e) Laggards are conservative and resist change and are thus the last to do so. According to this model, change can only occur in a stepwise manner, that is, innovators can only convince early adopters, early adopters can only convince early majority and so on until the laggards finally follow suit.

To grasp the interplay between the influence of social norms and individual readiness to change, we found the model of Shell-Duncan useful, particularly because it adds a perspective on decision-making power and the significance of external factors, such as policies and laws. She differentiates between five positions: willing and unwilling abandoners, contemplators, and willing and unwilling adherents. An unwilling abandoner, for example, is a person who would prefer that FGC continues but is unable to do so because of external factors, such as resistance from more powerful decision-makers or fear of legal repercussions. An unwilling adherent would likewise have been overpowered by other decision-makers, as illustrated in the introductory story.

Whereas these theories were developed and have been used mainly in countries where the topic of the study constitutes the local social norm, migrants in Norway live in a context where FGC is both legally and morally condemned by the host community as well as being increasingly questioned and abandoned by practicing migrant populations. Applying these theories in the context of migration may thus require further refinement and expansion of the factors that need to be considered. In the introductory story, for example, the circumcision of the girl was arranged by her aunt against the will and without the knowledge of the girl’s parents, which may have been facilitated by her father’s absence. Furthermore, the practice was in accordance with the social norms and values of the girl’s country of origin and was partially motivated by the girl’s imminent migration to a Western country. The aunt’s decision might further have been facilitated by the existence of a medical clinic where the procedure could presumably be done more safely in the transit country. Hence, traditional norms in the country of origin, changing decision-making groups and decision-making power due to migration, on one hand, and the legal issues and social norms in the country of intended migration influenced decision-making. In this article, we intend to further examine the nuance in these theories of change to better grasp processes of change related to FGC in the context of migration.

### FGC Among Somalis and Sudanese at Home and Abroad

Somali migrants were selected for the study because they constitute half of the 17,300 girls and women estimated to have undergone FGC prior to immigration to Norway (Ziyada et al., 2016). Sudanese migrants were selected because of major similarities in their FGC practices (Abdalla, 1982; Boddy, 1989), although they constitute a minority (3%) of those affected in Norway. FGC is almost universal among girls and women in both Somalia and Sudan, with a prevalence of 98% and 87%, respectively (UNICEF, 2016). Infibulation is the dominant form of FGC in both countries, and the closed vulva is regarded as evidence of the woman’s virginity and virtue. This closure has to be partially opened at marriage to enable sexual intercourse and, when performed by the husband, is commonly perceived as a test of his virility (Johansen, 2017). Further opening is usually necessary at childbirth. In Sudan, women are routinely reinfibulated after childbirth to recreate a tight vaginal introitus considered necessary to ensure male sexual pleasure (Almroth-Berggren et al., 2001). Routine reinfibulation has not been reported in the Somali population, where wounds are commonly said to be left to heal naturally (Johansen, 2016, 2017).

Despite the stronghold of traditional FGC-norms in both Somalia and Sudan (Bedri et al., 2019; Powell & Yussuf, 2018), international norms have been known for decades in both countries (Boddy, 2007; Lunde & Sagbakken, 2014). However, early initiatives targeted mainly infibulation (Abdalla, 1982; Boddy, 2007), which may have contributed to the current support for a change to a less extensive form of FGC rather than full abandonment of the practice (Crawford & Ali, 2014; Elmusharaf et al., 2006). This new type is commonly referred to as sunna circumcision, a term otherwise used to designate religiously recommended practices in Islam. A major motivation for the change of type is to mitigate the health risks associated with infibulation (Bedri et al., 2019; Powell & Yussuf, 2018). However, while there is an association between the anatomical extent of FGC and related health complications, there is no agreement about the physical extent of sunna circumcision, as the term has been used to denote procedures that are encompassed by all of the four types of FGC outlined by WHO (Crawford & Ali, 2014; Elmusharaf et al., 2006; Johansen, 2019): Type I—removal of part or all of the clitoral glans and/or its prepuce; Type II—removal of part or all labia minora, often with the clitoral glans and/or its prepuce; Type III—infibulation, that is, cutting and apposition of labia minora and/or majora to form a seal of skin that covers most of the vulva, usually following the removal of tissue from the clitoris and labia; and Type IV—any other procedure that may harm the external genitalia but that do not include tissue removal (World Health Organization, 2020). The potential health risks of sunna circumcision will thus vary with the physical procedure performed in its name.

FGC practices and discourses among Somali and Sudanese migrants both parallel and differ from those in countries of origin. The major deviation is a significantly stronger rejection of infibulation and major abandonment of all types in diaspora (Gele, 2013; Wahlberg et al., 2019). Despite these changes, studies have found significant trends of continuity, including adherence to underlying values, resistance against defibulation and acceptance of sunna circumcision (Alhassan et al., 2016; Gele, 2013; Johansen, 2019).

## Method

To gain insight into various positions regarding FGC, we recruited study participants with various migration experiences, lengths of stay in Norway, education levels, employment, and marital experiences. To conduct purposeful sampling, we used a snowballing method with different starting points, including both authors’ personal and professional networks, and direct contact with persons engaged in FGC interventions. A total of 23 women (15 Somali and eight Sudanese) participated in in-depth interviews between fall 2017 and summer 2018.

The interviews explored participants’ background, childhood and family situation, exposure to FGC discourses, social networks, and marital strategies and ideals. Interviews were conducted in a place chosen by the research participant, including private homes and meeting rooms at workplaces, libraries, and hotels, and lasted an average of 2 hours. Some participants (three) were interviewed twice to further explore elements from the first interview or because the first interview was interrupted. Two women were interviewed together upon their request.

All the Somali women and one Sudanese woman were interviewed by the first author, a medical anthropologist by training, and the interview was conducted in Norwegian English or with the support of a Somali interpreter (four interviews). Seven of the Sudanese informants were interviewed by the second author, a medical doctor with a master’s degree in public health, and the interviews were conducted in Arabic and English. Interviews were either tape recorded and transcribed (seven interviews) or careful notes were taken on the spot and written out immediately afterwards (16 interviews). The method of recording varied with the situation, the wishes of the participant, and the interviewers’ familiarity with the method.

Data analysis started during data collection and included repeated reading of transcripts to become familiar with the data, explore patterns and identify eventual needs for further investigation. Finally, the first author coded the data manually according to major themes and topics. Preliminary findings were subsequently presented for discussion in a group of five other Somali and Sudanese women. Our understanding was further complemented by insights gained through participant observations in various settings, including social encounters and workshops. We also engaged in informal conversations with approximately 50 other people, including Somali and Sudanese women in Norway; approximately 10 onward male and female migrants of Somali and Sudanese origin who had resettled in Canada, Kenya, the United Kingdom, and Sudan; and approximately six people of various origins working with FGC in Somali and Sudanese nongovernmental organizations or governmental institutions in Norway, the United Kingdom, and Kenya.

All potential participants were provided with oral and written information about the study, respondents’ rights, and confidentiality measures before giving their oral and/or written consent. All data are anonymized, and potentially identifiable factors have been omitted or changed. Recruitment, interviews, and storage of data complied with ethical requirements provided by the Norwegian Centre for Research Data (NSD), who confirmed that the study is in line with the personal data act (reference no. 55641).

### Reflexivity

Building trust between the researcher and research participant is paramount in qualitative studies, particularly when studying sensitive topics. In this study, the positioning of the two researchers in terms of being perceived as cultural insider or outsider could be a significant factor (Johansen et al., 2020; Kusow, 2003). The first author was likely perceived as a cultural outsider, as she is a native Norwegian who speaks neither Somali nor Arabic. The second author was likely perceived as a cultural insider, as she is of Sudanese origin and a native Arabic speaker. Being perceived as a cultural insider with shared experiences of migration and being a part of an ethnic minority may help build trust. However, it can also prompt reluctance to share information contrary to traditional norms and values for fear of judgment and gossips. Being perceived as an outsider, on the other hand, might inhibit trust, as one can be expected to share the judgmental attitude dominant in the Norwegian majority. However, it can also increase trust in sharing information that could be negatively judged within the community and reduce the fear of gossip.

Nevertheless, our experience was that our positions as cultural outsiders and insiders seemed to be counterbalanced by more personal factors. The first author encountered positive feedback regarding her more than 20 years of engagement with FGC and close contact with Africa and African migrants in Norway. Many expressed sentiments that her multiple visits to Somalia and Sudan, marriage to a man of African origin, and three daughters made them trust that she was on their side and could better understand their situation. For the second author, her Western dressing style and medical education may have contributed to improving trust that she would not be judgmental of deviance from the social norms perceived to be dominant in Sudanese networks in Norway.

Furthermore, both authors used several methods to build trust, including carefully outlining the codes of conduct of research. The interview guide was also designed with this in mind, being open-ended and flexible, giving participants a high sense of control of what information to share. In addition, the use of snowballing for recruitment was selected to help transfer trust. Finally, the researchers’ engagement in informal conversations—sometimes a shared meal and other joint ventures prior to the interview itself—was experienced as helpful.

## Findings

### Characteristics of the Study Participants

The 23 study participants were between 25 and 59 years of age, were first-generation migrants, had lived in Norway between 1 and 28 years, and were living in eight different towns and villages across the country. Most of them were or had been married, and most of them had children. Their educational level varied from 1 year of Quranic school to PhD. Overall, the Sudanese women had higher education levels than the Somali women, with several holding a university degree in contrast to only one Somali woman holding a university degree. Several of the Somalis had, however, completed vocational training. The participants had come to Norway as refugees (half of the Somali women), as part of a family reunion (most of the Sudanese women and the other half of the Somali women), or for education purposes (some of the Sudanese participants).

In total, 21 participants reported that they had been subjected to FGC, of whom 18 reported infibulation and three reported sunna circumcision. However, two of the women claiming sunna circumcision reported extensive labial closure, and thus, the procedure should probably be classified as Type III FGC. The two women with no FGC were of Sudanese origin. Most of the informants’ daughters born in the country of origin had undergone FGC, whereas none of those born in Norway had undergone FGC.

### Maneuvering Between Traditional and International Meaning and Norms

FGC decisions made after settlement in Norway included seeking health care services, marital strategies, meaning-making, and engagement with the topic. As indicated, women’s positioning and decision-making required a maneuver between diametrically opposed meanings of FGC, as either a prerequisite for and evidence of female virtue, or the ultimate expression of oppression of women’s sexual and reproductive rights. Positioning between these norm sets was further found to be associated with their relationships to the different social networks upholding these norm sets.

The theory of FGC as a social convention initially relied heavily on a perception of FGC as a prerequisite for marriageability, which again assumes ethnic endogamy. Although this aspect has lately been nuanced following critical assessment (Mackie & LeJeune, 2009), it is considered important in both Somalia and Sudan, where infibulation is further closely interlinked to patrilineality as the governing social principle (Boddy, 1989; Talle, 1993). We explored whether changing practices of and attitudes toward FGC in the diaspora were interrelated with changes in marital ideals. To explore this, we included questions about marital ideals and experiences as well as purposefully recruited four women who had exogamous marriages.

In the following sections, we will describe the ways in which participants maneuvered between traditional and international norms regarding FGC and different social networks and how this relates to their positioning along a continuum of readiness to change. Informed by the theories of change outlined above, we draw a continuum ranging from women who are eager to abandon FGC and adopt international norms to women who support the continuation of FGC and its traditional cultural meaning. We grouped research participants into three broad categories, which we refer to as “rebels,” “contemplators,” and “adherents,” indicating their position along the continuum of readiness to change.

### The Rebels: The Drivers of Abandonment

Women classified as rebels rejected both infibulation and sunna circumcision. They also rejected traditional norms and values underlying the practice, including the idea that virginity could or should be proven. Instead, they embraced international norms, comprising a perception of FGC as a violation of their human rights, including the right to bodily integrity and control of their sexual life. As a practical consequence, they not only refrained from cutting their daughters but also eagerly embraced the opportunity for medical defibulation, perceiving it partly as a way of undoing FGC. Many also diverged from traditional norms in other ways, such as Western attire and/or not wearing a veil, entering an exogamous marriage, and engaging in activities promoting women’s sexual and reproductive rights and gender equality, including FGC abandonment. Furthermore, although they all kept in contact with their families and identified as Somali or Sudanese and devout Muslims, they stood by their choices despite negative social sanctions from family members and other co-nationals. They generally prioritized social networks with likeminded women and men independent of ethnic and national background over family and co-nationals.

They did not, however, experience this position as easy and carefree. In contrast, many rebels expressed a sense of being torn between a longing for belonging and acceptance in their ethnic communities and a strong desire for individual freedom, particularly freedom from what they perceived as restrictive traditional social norms. A typical example was a Somali woman who considered her refusal to veil a protest against the strict Somali norms, and simultaneously, an expression of her religious devotion: “*I love God, but that has nothing to do with what I wear on my head. So, one day, I threw away the hijab as a protest against the strict social control.”* Thus, in contrast to a common trend of increased covering following increased religious knowledge and devotion after migration (Liberatore, 2016b; Talle, 2008), this woman had adopted more personal religiosity.

This woman, who had come to Norway as a young adult, recalled being rebellious since childhood. She considered her rebellious streak to be caused partly by her experience with FGC, a traumatizing repeat infibulation at the age of 7 years. This was followed by a feeling of being exposed and different because of her FGC, as the practice was not common in the Arab country in which she was born and raised. However, this country was conservatively Muslim, and she had contested its strict gender roles since a young age. She laughingly recalled how she had tied the veil of the school uniform in a way mimicking local men’s headwear. She also presented her migration to Norway as motivated by her desire to escape restrictive gender roles. Nevertheless, she initially tried to fit in with the Somali community in her Norwegian hometown through dressing, socialization, and religious engagement. After a few years, however, she again felt confined by community social norms and started to change. First, she stopped veiling. Then, she entered a relationship with a native Norwegian man. Upon proclaiming their intention to marry, her family, spread across a large number of countries, was horrified. They had bombarded her with phone calls to argue her out of it. One day, her brother, who lived in another European country, showed up at her door:

*At first, I was scared to open the door to him because he had yelled at me so much for weeks over the phone, calling me horrible names. Then, I opened, and he locked me into a room for many days. [ . . . ] But my dad was amazing. He called and supported me. He said nobody could treat his daughter this way. My mother also turned from being angry to giving in. So now I have my freedom.*

This woman’s story was typical in the sense that while many had experienced family pressure to comply with traditional norms, most rebels were simultaneously supported by other family members. In addition, she encountered negative social sanctions in the local Somali community, mainly in the form of social exclusion and gossips. She understood her exclusion to be partly motivated by a fear that other young Somali women could follow in her footsteps: *“Somali families cross to the other side of the street when they meet me. I think they are scared I will have a bad influence on their daughters.”* She thus felt that her noncompliance with traditional norms not only compromised her personal reputation but was also perceived as a risk to the moral integrity of other young Somali women.

However, despite her rebellious attitudes, she struggled with letting go of the embodied meaning of her infibulation. Although her husband’s Norwegian origin meant that her infibulation was of no importance for marriageability, she resisted seeking medical defibulation for more than a year into the marriage. Her infibulation was intimately tied up with her image of herself as a good and proper woman. For her, as for many other women, FGC was not only important as a social convention or normative expectation but also as a lived bodily experience. However, her experience contradicted a common perception of exogamous marriages as a result of securing an endogamous marriage due to failure to preserve virginity, as found in other studies (Johansen, 2019).

Another rebel who had an exogamous marriage was a Somali woman in her forties. Like the woman above, she expressed a sense of being torn between the sadness of exclusion from the local Somali community and fear of its social control: *“I am divorced from my community, I miss it, but I also fear social control. Now I only take part in funerals and some parties.”* She encountered negative comments or expressions of concern from co-nationals regarding the religious upbringing of her children, as her Norwegian husband had not converted to Islam. To mitigate social repercussions affecting her daughters, she physically distanced herself from the Somali community by moving to a small town with no other Somali residents. Although she encountered other Somalis through her volunteer engagement, she socialized mainly with likeminded women across a variety of ethnic groups.

The other two women who had exogamous marriages—including a Somali woman in her fifties and a Sudanese woman in her forties—reported fewer negative reactions from family and co-nationals than the women above. They both thought this was because of this being their second marriage; hence, they had already proven their virginity and virtue through their first marriage with a co-national.

Three of the women who had exogamous marriages described actively searching for a non-Somali/Sudanese partner. They had preferred a native Norwegian husband because they expected this to ensure a more equal marriage. The rebels who had married co-nationals had similar wishes and to achieve this had searched for a “modern” man, commonly by virtue of education and life in diaspora.

The visible rebellious acts of these women, such as attire, marital choices, place of settlement, and social engagement, were met with various levels of negative social sanctions and social exclusion from co-nationals. Other rebels seemed to be able to “pass” as conformists. One example was a Sudanese woman in her fifties. Since experiencing her own FGC as a violation and intrusion on the very body part she was told to protect and keep private, she had rejected the practice by refusing to participate in FGC celebrations while still living in Sudan. As an adult, she had taken care that her daughters were not cut as well as working to convince others to follow suit. Although she had married a fellow Sudanese person, she laughingly hoped her daughters would not follow suit.

The rebels presented their position as an active choice, brought about by their personality, upbringing, experience, and critical reflection on their culture. They perceived themselves as strong and self-assertive and emphasized personal freedom over belonging and social acceptance. In addition, all of the rebels had higher education and were financially independent. Thus, they may have been more willing to act on their resistance to FGC because they had a higher perceived power to do so. In addition, they had developed cross-ethnic social networks with likeminded women and men that supported their engagement in promoting international norms and FGC abandonment. Thus, there were fewer social costs.

Nevertheless, the rebels’ rejection of the traditional meaning of FGC and the embrace of international norms did not lead them to assimilate into dominant Norwegian culture or any other ethnic community. Quite on the contrary, most of them engaged in voluntary work within their ethnic community or had plans to do so, often to promote women’s sexual and reproductive health and rights. This engagement seemed partly a way for the women to reconnect with their respective ethnic communities, as indicated by a Somali woman: *“One day I will go back to Somalia and help my girls. They need my help to escape FGC.”* Her statement also emphasized ethnicity over nationality, as she was born and raised in an Arab country and had never visited Somali regions. Thus, the rebels seemed to perform a sort of cultural brokerage, making efforts to change their culture from within. In this process, they negotiated their position from one of feeling pressure to comply with traditional norms into one that set the scene to spur change toward international norms.

### The Contemplators: Ambivalence Toward FGC

The contemplators—positioned along the middle of the continuum—expressed ambivalence of various aspects of FGC, including its underlying norms. They rejected infibulation but simultaneously expressed ambivalence toward premarital defibulation, reinfibulation, and sunna circumcision, and they rarely challenged traditional values associated with FGC. Their ambivalence varied with regard to the relative weight they put on personal opinions and perceived social norms, but a sense of need to comply seemed to weigh heavily in their decision-making. A typical example is the way they resisted premarital defibulation because it would contradict the perceived social norm of virginity proven through an intact infibulation, despite expecting such a procedure to improve their well-being and reduce eventual FGC-related complications.

The perceived need to demonstrate virginity though infibulation was further linked to their perceived expectation of ethnic endogamy, as FGC is unlikely to be expected by a man with a different cultural background. Some of the Somali participants even emphasized clan endogamy, which led some of the women to cross the Norwegian borders to find suitable partners. One of them had spent a year visiting relatives in various countries in the pursuit. During this time, she had reconnected with a male acquaintance she knew through several family holidays as a child. After this last visit, the two remained in contact, and approximately 1 year later, the man proposed. The woman spoke warmly and laughingly about his careful balance between traditional and Western norms when politely asking her parents for her hand, but without the customary accompaniment of relatives, gifts, or a suitable outfit.

Prior to their wedding, the groom had asked about her FGC status. She initially considered the question intrusive but relented when he explained this as a concern for her well-being and potential need for surgical defibulation. Only then did she reveal that she was not fully closed, as the closure had partially reopened during the healing period. She was relieved when he accepted her state. Relatives and friends attending the wedding had nevertheless bestowed her with advice on how to cope with the pain of sexual initiation. Their advice terrified her. After a painless and effortless sexual initiation, she realized that the advice she had received was based on an assumption that she was infibulated. Manual defibulation is commonly a painful procedure involving tears and wounds in the infibulated seal of skin (Johansen, 2016).

We categorize this woman as ambivalent because of the way in which her resistance against infibulation was counterbalanced by strong trends of continuity in associated cultural values. This included the importance of premarital virginity and ethnic endogamy. She socialized only with other Somalis, was reluctant to discuss the practice within this network, and dressed in the “neo-traditional” Somali attire (*jilbaab*), which seems to constitute a dominant social norm among Somali migrants (Liberatore, 2016a; Talle, 2008). Thus, she carefully balanced traditional and international norms and expectations.

Another contemplator was a Somali woman in her mid-twenties. Her negative attitude toward FGC implied a decision to refrain from taking her daughter with her when visiting family in country of origin. She feared they would enforce her daughter’s FGC, a fear based on her own experience of being infibulated at the age of 8 years: “*I would never dare to bring my daughter to Somalia. My grandmother had me cut even when my mother was against it. If I say anything, they say you have a Norwegian tradition.”* We also see how she felt even more powerless in standing up to her family wishes precisely because of her current residence in Norway because her arguments would be discredited as Norwegian. Thus, although life in the diaspora had made her decide against FGC, it had simultaneously undermined her opportunity to stand up against the practice in country of origin.

This sense of powerlessness vis-à-vis the wishes of her family was also the reason she had never contemplated marrying outside her clan, which she expected them to object to. She felt this tradition had been strengthened following recent years clan-based conflicts in Somalia. She also supported some of the traditional underlying norms, especially virginity, and thus could not envisage premarital defibulation. Neither had she considered defibulation at marriage, as this would have deprived her husband of a chance of proving his virility, as she said: *“It (defibulation) would be like accusing him of being unable to do his job.”* This woman’s ambivalence thus comes from a combination of personal conviction and a sense of vulnerability vis-à-vis social norms upheld by her family in country of origin.

Most contemplators expressed a sense of ambivalence toward sunna circumcision in the sense that while they did not promote or actively support it, they did not find it problematic. Most referred to sunna circumcision as a harmless procedure or at least significantly less harmful than infibulation. None of the contemplators expressed a wish or a plan to subject their daughters to sunna circumcision, mostly due to Norwegian law. When taking place in the country of origin, however, they considered it less problematic. One example is a Somali woman who initially expressed pride in having protected her nieces in Somalia from FGC, but later in the interview revealed that this referred to them being subjected to sunna circumcision rather than infibulation. Her outline of the physical extent of sunna circumcision corresponded to WHO Type II and thus hardly a minor procedure.

Although the position of the contemplators along the continuum differed, they all presented their position as a careful balancing act between personal preferences and social norms. They expressed both a relatively strong sense of dependency on social acceptance from co-nationals, as well as personal support for the traditional norms and values linking FGC to virginity, virtue, and morality.

Several interlinked factors could contribute to the contemplators’ sense of vulnerability vis-à-vis the perceived social norms in their ethnic community. They generally had fewer resources for independence in the form of formal education and employment than the rebels. Some of them also highlighted young age and living parents as a disempowering factor. Furthermore, they mainly socialized with co-nationals. These factors may be interlinked. Women without education and/or work often expressed a strong sense of dependency on their ethnic network for financial, practical, and emotional support. Given the experience of exclusion and harassment experienced by the rebels, the limited social capital may reduce the contemplators’ willingness to deviate from social norms of the ethnic community, as the price would be experienced as much higher for them than for the rebels.

### The Adherents: Resisting Change

Only one of the participants openly expressed support for FGC and could thus be classified as a clear adherent. This was a Sudanese woman whose family had abandoned FGC in the 1960s. Growing up in Sudan, however, she had suffered so much from peer pressure and harassment that she had managed to convince her mother to have her circumcised. Now, many years later as an adult woman living in Norway, she supported sunna circumcision because she understood it to be recommended by Islam. Still she had no intention to cut her daughter, partly because she knew it to be illegal in Norway, but more importantly due to the lack of clarity about the religiously correct sunna circumcision. She thus feared that FGC providers would not know how to correctly conduct the procedure. This adherent would thus correspond to Shell-Duncan’s category of unwilling abandoner.

Despite only one clear adherent of FGC for girls, many of the Sudanese contemplators expressed some forms of adherence, including support for reinfibulation. One example is a woman who expressed relief that she had given birth by C-section, thus escaping the need to return to Sudan for reinfibulation. Furthermore, reinfibulation was commonly perceived as a comprehensive procedure that recreated a vaginal orifice similar to that of an unmarried woman. In contrast, resuturing back to a predelivery vaginal opening was generally not considered a form of reinfibulation and thus found to be even a standard procedure, even though it is illegal in Norway.

Furthermore, many participants shared stories indicating that being uncut, or medically defibulated, would be socially unacceptable in countries of origin, thus making it impossible to resettle in country of origin after premarital defibulation. It would not only pose a problem at an eventual marriage but was expected to be known regardless, for example, through the sound or time of urination or behavioral changes. One of the younger women laughingly recalled how friends and relatives had challenged her jokingly for having excess sexual urges because she was uncut when she was actually trembling due to cold weather during a holiday in Somalia.

The study participants also shared numerous stories of the opinions and behaviors of others in country of origin, onward migrants, return migrants, and migrants in other countries. A Sudanese woman in her thirties, for example, spoke about a friend who, after raising her daughter in Norway, had migrated to another European country. Then, she was planning to subject her daughter to FGC in her country of origin. Two other women, a Sudanese woman in her fifties and a Somali woman in her thirties, reported friends living in European countries who had recently taken their daughters to their country of origin for FGC. One of the rebels shared a story of an acquaintance who had returned to Somalia for employment with her teenage daughter but had chosen to return to Norway after a few months because her daughter had experienced so much harassment that she had begged her mother to be cut. Onward and return transnational migration, found to be conducted by 15% of Somalis who have gained Norwegian citizenship, might thus pose a risk of changing back to FGC (Vassenden, 2020).

Secondhand stories of FGC are likely less reliable than those told in first person. However, their sheer number and the eagerness with which they were told suggests that they may constitute a significant part of a transnational discourse on FGC decision-making in the context of migration that helps women navigate their FGC decision-making.

## Discussion

In this section, we will discuss the theories of Mackie’s, Rogers’, and Shell-Duncan’s suitability in assessing readiness to change in the context of migration. We will start with a reflection on similarities and differences between our categories and those of the selected theories. Then, we will investigate similarities and differences in the process of change. Last, we explore the significance of meaning-making according to traditional or international norms and the choice and significance of various social networks.

Comparing our categories of positioning with those of Rogers’ and Shell-Duncan’s, we find some differences associated with migration that may be important to consider. Our category of rebels is similar to Rogers’ category of early adopters and Shell-Duncan’s willing abandoner. The rebels had changed most substantially, abandoning all forms of FGC, supporting defibulation, resisting reinfibulation, and adopting international normative understanding of the practice. We found a close connection between their dramatic change and their ethnically diverse but normatively supporting social network.

Social convention theory builds on a perception of social norms in social reference groups as the main factor for continuation or change. However, the delineation of the social reference group seems to differ fundamentally between countries of origin and countries of migration. In countries of origin, social reference groups are commonly delineated with ethnicity and locality as proxies (Wander & Shell-Duncan, 2020). In the diaspora, however, locality may have little or no relevance, as patterns of residence are often driven by external factors such as settlement policies and study and work opportunities. Ethnicity may also be of less relevance, as migrants are surrounded by natives as well as numerous other migrants with various ethnic and national backgrounds. Finally, significant others, such as family and relatives, including those traditionally holding substantial decision-making power, are often spread across the globe. It is thus not clear what would constitute a significant reference group in diaspora, and our findings indicate an element of choice: particularly for the rebels, who distanced themselves from their own ethnic networks and sought networks with likeminded others, thus creating their own reference group. To what extent they prioritized these networks because of their rebellion, or it was these networks that encouraged or facilitated their rebellion, may vary. However, the rebellious strike most rebels recalled since childhood and their distancing from their ethnic communities suggest that internal drive or personality may be a major driving factor in their quest for change.

Furthermore, rebels not only abandoned and reinterpreted FGC in line with international norms but also engaged actively to influence others along. Despite social exclusion from their ethnic communities and prioritizing the social network of likeminded men and women, almost all of them were actively engaged in making efforts to change the cultural meaning of FGC within their ethnic communities through voluntary engagement.

We would not, however, liken the rebels to Rogers’ category of innovators, which we would reserve for some of the first migrant women who targeted FGC in Norway approximately 20 years earlier (Johansen, 2006). These women were portrayed as heroes in the Norwegian host society but often experienced hash reactions from their ethnic communities. Some of them periodically needed police protection, and many of them were said to be considered traitors to their ethnic communities. The women in this study, in contrast, worked in a more low-key manner, believing this to be most efficient at this point in time. Their emphasis on low-key action was partly grounded in the public stigma of FGC that has particularly affected Somali migrants and contributed to increasing skepticism and silence in dealing with the issue (Johansen, 2019; Talle, 2008).

The contemplators correspond to Shell-Duncan’s category with the same name as well as Rogers’ early and late majority, which constitutes the largest group. Their ambivalence was evident in their rejection of some aspects of FGC and acceptance or support of others. On one hand, they all rejected infibulation and had no plans to cut uncut daughters. On the other hand, they generally rejected premarital defibulation, accepted sunna circumcision in country of origin, expressed support for underlying cultural values, were reluctant to engage against FGC, and the Sudanese contemplators also supported reinfibulation. Women varied in the extent to which they presented their views as personal convictions or a sense of need to comply to their overall co-ethnic social network, but the latter seemed often to be considered as most important. One example is that while they grounded their rejection of infibulation in their health adversities—adversities many considered as making the practice counter to Islam—they were inclined to consider premarital defibulation as a form of social suicide. A recent study on health-seeking behavior among Somali and Sudanese migrants in Norway found a similar pattern (Ziyada et al., 2020). Our findings thus support suggestions that adherence to and ambivalence about FGC is not necessarily due to ignorance of the pain and health risks involved but rather that some perceived this as the price you have to pay for social acceptance (Hamed et al., 2017; Johansen, 2002; Wander & Shell-Duncan, 2020). The contemplators seemed to be more vulnerable to this, as they socialized mostly within their own ethnic group. In the context of migration, locality may be of limited relevance as a proxy of the reference group, whereas ethnicity may be important for people who choose or feel compelled to relate to their ethnic network as their main reference group.

Our category of adherents is similar to Shell-Duncan’s category of unwilling abandoners and Rogers’ laggards. However, the major contextual differences between the country of origin and the country of migration imply that these positions pose differing limitations and opportunities in the diaspora. In Norway, the performance of any form of FGC, including infibulation, sunna circumcision, and reinfibulation, is impossible due to legal regulations. Thus, whereas being a willing adherent in country of origin could be rather simple, in the diaspora, it would imply serious risks of legal prosecution, lost custody of one’s children, expulsion, or a need for resettlement in country of origin. Reinfibulation, however, seems to be accessible through travel and hardly causes a risk of legal prosecution if conducted abroad.

Despite a lack of evidence of support for infibulation among migrants in Norway, some participants firmly believed that some of the elderly and more conservative community members supported the practice but that they kept quiet for fear of legal repercussions. While they believed these people to abstain from subjecting the girls in the family to FGC for the same reason, they feared they would take up the practice if they were to resettle in a country where this would be possible.

Furthermore, we argue that the process of change differed from those outlined in the employed theories of change in the context of migration. FGC has largely been abandoned in Norway in the sense that there are very few cases of young girls who are raised in Norway and subjected to FGC. This indicates that people generally stop practicing FGC in the diaspora, irrespective of their opinion. This contrasts with the employed theories of change, as they imply that the first to change is attitude, and actual abandonment follows next. This distinction is most clear from the expectations of change in country of origin according to the theory of FGC as a social convention, as one here expects change will occur only once a significant group has agreed on abandonment. Neither did change occur in one group at a time, as suggested by Rogers. Rather, we found changes all along the continuum, though with varying speed, intensity, and content. Furthermore, our only adherent illustrates that change may go both ways, as she supported the practice despite her family having decided to abandon it decades ago. Such fluctuations and moves in both directions over time have been recorded in other studies (Nypan, 1991; Wander & Shell-Duncan, 2020).

Among the factors found to hinder or drive change, we found meaning-making and significant social networks to be of major importance. Our intention in exploring meaning-making in terms of the extent to which women make sense of FGC according to traditional or international norms was to explore the potential role of meaning-making in processes of change. One of the arguments for the suitability of the theory of FGC as a social convention in interventions is that it does not require cultural changes or changes in meaning (UNICEF, 2005). If the social convention changes to not expect FGC, the major motivation of the parents of securing the future of their daughter will no longer require FGC. Along with the emphasis on FGC as a coordinated social norm, the theory assigns minimal weight to cultural meaning. In a recent revision of the theory, however, Mackie and colleagues suggest FGC abandonment to be more difficult in communities associating FGC with ideals of morality and virtue (Mackie & LeJeune, 2009). As we have seen, this is very much the case for Somali and Sudanese FGC traditions. It is thus worthwhile to explore the significance of meaning-making for the process of change among migrants from these countries.

Our findings indicate that meaning may play a major role in women’s readiness to change. We saw how the rebels’ rejection of traditional meaning and embrace of international norms were major drivers in their quest not only to abandon FGC but also to drag others along as well as to work for women’s sexual and reproductive health more broadly. In contrast, the contemplators often expressed a sense of constraint against change. For example, they felt unable to seek needed health care because they perceived it as undermining the value of virginity and virtue. Furthermore, we may regard the acceptance of sunna circumcision as a form of continuity in the traditional meaning of FGC as a vehicle of control of women’s sexuality. This is linked to ideas that consider infringements of the clitoris to reduce women’s libido and thus the risk of illicit sexual engagement (Johansen, 2007). However, the acceptance of sunna circumcision is also linked to a new form of religious legitimacy, as many consider sunna, in contrast to infibulation, as religiously condoned (Crawford & Ali, 2014; Johansen, 2019). This new religious legitimacy may at the same be considered as a continuation of FGC as a form of sexual control in that it is believed to facilitate compliance with religious sexual norms that to a great extent coincide with traditional norms, emphasizing virginity and virtue.

The other factor found to play a significant role in women’s readiness to change was their social networks, particularly in terms of networks that differed in terms of meaning-making. We saw how the rebels prioritized social networks that shared their attuning to international norms and felt less obliged by the social norms in their ethnic communities. In contrast, the contemplators who mainly socialized with members of the same ethnic group shared with them stronger adherence to traditional norms and expressed a stronger sense of vulnerability and dependency of securing social acceptance through conforming to these values. This finding is supported by general migration studies, finding that while close ethnic networks may foster solidarity and mutual support, it is also associated with a stronger experience of social control and pressure to conform (Vacca et al., 2018). However, as we saw, social networks may have an element of choice in the diaspora, which was especially reported by the rebels, whereas contemplators and adherents more often presented their social networks as a given.

The significance of social networks is thus further linked to decision-making power. The rebels’ choice to breach some of the social norms in their ethnic network despite negative sanctions may not (only) be due to their stronger conviction but also because of their higher or broader social capital. Overall, the Somali rebels had higher levels of education and employment in addition to social networks beyond their own ethnic group and family than the contemplators. This would reduce the social costs of breaking traditional norms. Similar changes have been found in urban and multiethnic communities in Africa (Cloward, 2015; Shell-Duncan et al., 2011). The contemplators, in contrast, commonly had less education and employment and a more ethnic homogeneous social network. Thus, they had a perception of fewer exit options. Among the Sudanese, the difference between the groups was only in terms of social networks, whereas there was no systematic difference with regard to education and employment. This difference corresponds with findings from their respective countries of origin (UNICEF, 2013).

It may thus be useful to analyze the role of social capital differently in the context of migration and country of origin. A study from Senegal and Gambia suggested that FGC bestows women with social capital (Wander & Shell-Duncan, 2020), whereas in the context of migration, social capital may facilitate FGC abandonment.

## Conclusion

In this article, we explored readiness to change regarding FGC and the factors that influence it among Somali and Sudanese migrants in Norway. We defined FGC decision-making broadly, including physical, cultural, and social aspects of the practice. The physical aspects included perceptions of different types of FGC and its management, including deinfibulation and reinfibulation. Cultural factors refer to meaning-making, particularly whether FGC is understood in terms of traditional or international norms. We found this to be influenced by social networks, particularly the extent to which women socialized mainly with co-nationals or likeminded people irrespective of ethnic or national origin.

Although all women showed elements of change, the extent, content, and intensity varied. Based on three different theories of change, that is, Rogers’ theory of diffusion of innovation, Mackie’s theory of FGC as a social convention, and Shell-Duncan’s model of readiness to change, we grouped women into three main categories along a continuum of readiness to change. At the one end, we placed the rebels, who resisted all forms of FGC, embraced international norms, and prioritized social networks with likeminded men and women. The contemplators, situated in the middle of the continuum, expressed ambivalence both toward some of the physical aspects of FGC, such as sunna circumcision and deinfibulation and reinfibulation, upheld most of the traditional norms and socialized mainly within their own ethnic group. At the other end, we placed our only adherent, who supported some of the values underlying FGC.

Based on these findings, we argue the need to complement existing theories of change adapted to countries of origin, with a more thorough investigation into meaning-making and social networks that are important for a better understanding of processes of change in the context of migration. Despite the general abandonment of FGC found, there could be a risk that some contemplators and adherents could revert to practice various forms of FGC if they resettle in a context where this was promoted and/or facilitated. This risk is real, as was highlighted in the many stories women told about acquaintances that had reverted to FGC after onward and return migration. It could also constitute a barrier for the global process of abandonment. We thus suggest that the rebels’ fundamental change, in that they also changed the normative framework for FGC meaning-making and actively engage against FGC, implies that they can play a pivotal role in driving profound and sustainable change both locally and transnationally.
